# CNGA3-Related Achromatopsia: A 10-Year Follow-Up

**DOI:** 10.1177/24741264251414135

**Published:** 2026-01-28

**Authors:** Haaris M. Khan, Fernando A.G. Sumita, Rony Carlos Preti, David Sarraf, Eduardo V. Navajas

**Affiliations:** 1 Department of Ophthalmology and Vision Sciences, University of British Columbia, Vancouver, BC, Canada; 2 Division of Ophthalmology, University of São Paulo Medical School, São Paulo, São Paulo, Brazil; 3 Stein Eye Institute, University of California Los Angeles, Los Angeles, CA, USA

**Keywords:** achromatopsia, disease progression, hyperreflective foci

## Abstract

**Purpose:** To describe long-term structural retinal changes in CNGA3-related achromatopsia using spectral-domain optical coherence tomography (SD-OCT) over a 10-year follow-up period. **Methods:** A single case was reviewed. **Results:** A 16-year-old girl with genetically confirmed CNGA3 mutations underwent annual SD-OCT imaging with concurrent assessment of best-corrected visual acuity (BCVA). Over the 10-year follow-up, BCVA remained stable; however, progressive foveal structural deterioration was observed. These included early external limiting membrane (ELM) hyperreflectivity and ellipsoid zone (EZ) disruption, followed by the development and enlargement of optically empty spaces, choroidal hypertransmission defects, and increasing hyperreflective foci. These findings were consistent with progression through a previously proposed OCT-based staging system for achromatopsia. **Conclusions:** This case demonstrates that CNGA3-related achromatopsia can exhibit clear structural progression on SD-OCT despite stable visual acuity, challenging the traditional view of the disease as stationary. SD-OCT is essential for detecting subtle but progressive foveal degeneration, and hyperreflective foci may represent an early marker of photoreceptor or retinal pigment epithelium compromise. These findings support further refinement and validation of OCT-based staging systems in CNGA3-related achromatopsia.

## Introduction

Complete achromatopsia is a rare inherited autosomal recessive disorder that affects approximately 1 in 30 000 live births worldwide.^1^ The disease is characterized by loss of cone photoreceptor function and typically presents with color blindness, pendular nystagmus, photophobia, reduced visual acuity (VA; approximately 20/80 to 20/200),^2^ and central scotoma.^2,3^ Diagnosis is based on a combination of clinical features, electroretinography (ERG), spectral-domain optical coherence tomography (SD-OCT), and genetic testing.^4^ Currently, there are no curative treatments available, and patient care revolves around symptom management and optimization of residual visual function.

Achromatopsia was traditionally thought of as a stationary disease; however, both human and animal models have illustrated that disease progression does occur.^5^ Serial OCT imaging reveals progressive structural changes, ranging from subtle disruption of the inner segment ellipsoid zone (EZ) to complete retinal pigment epithelium (RPE) atrophy.^5^ These morphological findings are often accompanied by worsening of VA.

Despite advances in our understanding of achromatopsia, longitudinal studies detailing the long-term progression of structural retinal changes remain limited. Therefore, we present the progressive structural findings of a patient with CNGA3-associated achromatopsia followed by her eye care team for 10 years.

## Case Report

A 16-year-old girl presented to the retina service complaining of blurry vision, pendular nystagmus, and photophobia in both eyes since birth. Her family history and past medical history were unremarkable.

**Figure 1.**
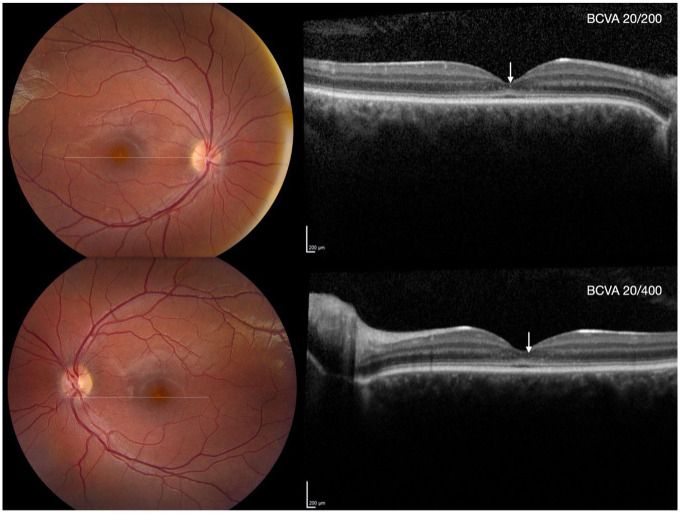
Baseline visit. Top left and bottom left: Color fundus photographs of the right and left eye showing mild yellow discoloration of the fovea. Top right and bottom right: Spectral-domain optical coherence tomography (SD-OCT) of the right and left eyes showing thickening of the external limiting membrane, flattening of the ellipsoid zone (EZ), and hyporeflectivity of the interdigitation zone in the center of the fovea. White arrows indicate hyperreflective foci in the outer nuclear layer. The distance between the EZ and interdigitation zone remained constant from the parafovea toward the periphery of the SD-OCT B-scan.

**Figure 2.**
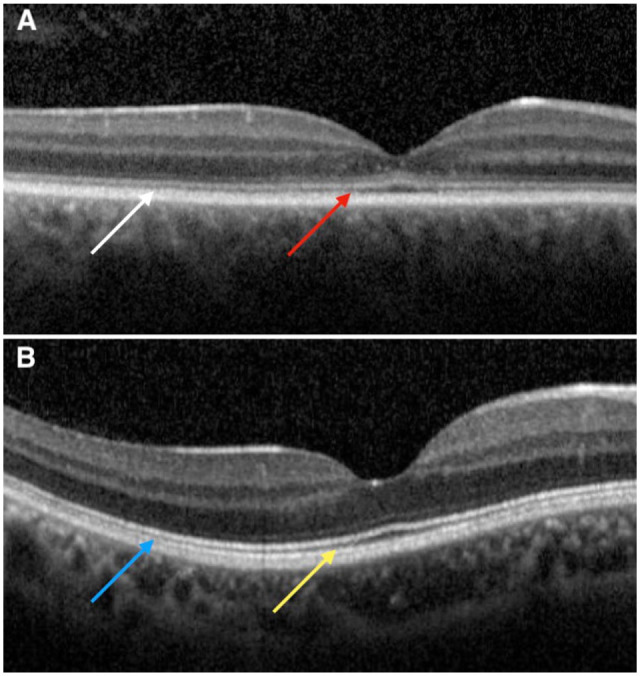
(A) Baseline spectral-domain optical coherence tomography (SD-OCT) demonstrating a constant distance between the interdigitation zone and the ellipsoid zone (EZ), as indicated by the red and white arrows. (B) SD-OCT image of a healthy control eye showing a greater distance between the EZ and interdigitation zone in the central fovea (yellow arrow) compared with more peripheral regions (blue arrow), illustrating the physiological decrease in this distance with increasing eccentricity from the fovea.

**Figure 3.**
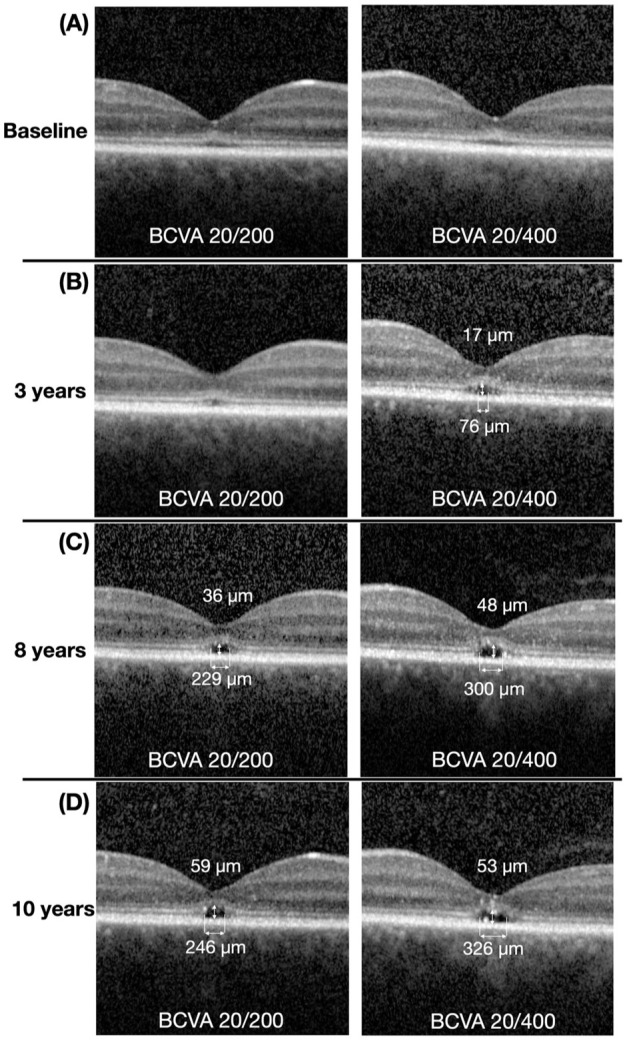
Progression over 10 years of follow-up on spectral domain optical coherence tomography (SD-OCT). Left row: right eye. Right row: left eye. (A) Baseline visit. (B) Three-year follow-up. (C) Eight-year follow-up. (D) Ten-year follow-up. Measurements in microns (µm) above and below the fovea represent the height and width of the optically empty space, respectively. Images reveal progressive central ellipsoid zone loss with the development and enlargement of a central optically empty space over time.

The best-corrected VA (BCVA), measured using the Early Treatment Diabetic Retinopathy Study chart, was 20/400 OD and 20/200 OS. The intraocular pressure was 11 mm Hg OD and 12 mm Hg OS. Color fundus photography revealed yellow discoloration of the fovea in both eyes. SD-OCT revealed disruption of the external limiting membrane (ELM) at the center of the fovea, along with hyperreflective foci in the outer nuclear layer (ONL) at the fovea. The interdigitation zone was attenuated, and the distance between the EZ and interdigitation zone remained constant from the parafovea toward the peripheral regions of the B-scan (Figures 1, 2, and 3A). The ONL thickness measured at the center of the fovea from the internal limiting membrane to the ELM was 28 µm OD and 35 µm OS, and the central macular thickness (CMT) was 112 µm OD and 122 µm OS.

Achromatopsia was diagnosed through genetic testing, which demonstrated heterozygosity for pathogenic variants in the CNGA3 gene [c.1557G>A, p.(Met519lle) and c.1641C>A]. The patient was subsequently monitored with regular follow-up visits and annual SD-OCT imaging. After 3 years, there was no change in BCVA, and SD-OCT imaging of both eyes showed mild disruption of the EZ, more pronounced in the left eye (OS > OD). A focal area of interdigitation zone discontinuity was observed at the center of the fovea in both eyes. This discontinuity was wider in the left eye, forming an optically empty space in the outer retina, measuring 76 μm in horizontal width and 17 μm in vertical height (Figure 3B). The ONL thickness was 44 μm OD and 36 μm OS, while the CMT was 129 μm OD and 125 μm OS.

Eight years after the initial visit, SD-OCT of the right eye showed mild EZ disruption and the presence of an optically empty space measuring 229 μm in width and 36 μm in height. This optically empty space subsequently enlarged to 300 μm in width and 48 μm in height (Figure 3C). SD-OCT of the right eye also showed hyperreflective foci within the EZ. In the left eye, hyperreflective foci were observed within the EZ and at the temporal margin of the optically empty space.

**Figure 4.**
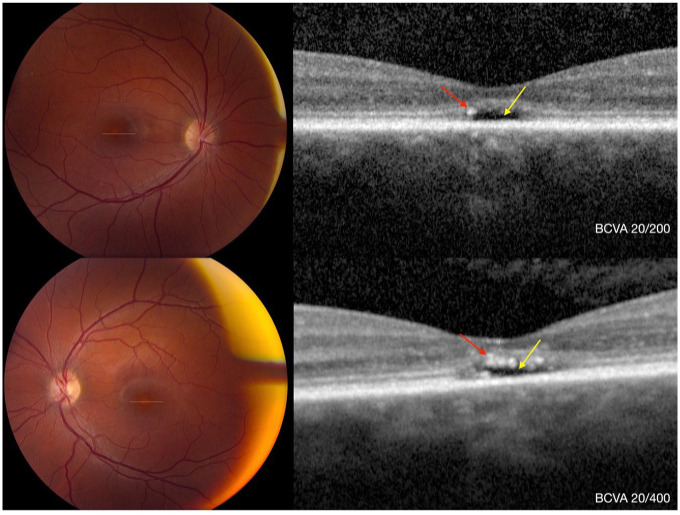
Ten-year follow-up imaging. Top row: right eye. Bottom row: left eye. Color fundus photographs of both eyes show persistent yellow discoloration of the fovea, similar to baseline findings (Figure 1). Corresponding spectral domain optical coherence tomography images demonstrate an optically empty space (yellow arrow) and hyperreflective foci (red arrow).

**Figure 5.**
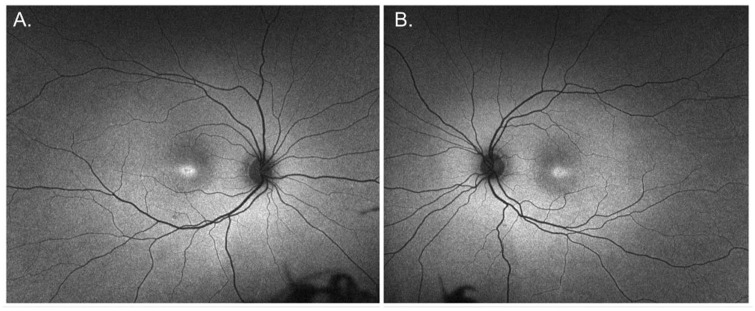
Ultra-widefield autofluorescence of both eyes at 10-year follow-up. (A) Right eye showing a ring of hyperautofluorescence with a small central area of hypoautofluorescence in the fovea. (B) Left eye showing a similar ring of hyperautofluorescence with a less pronounced central hypoautofluorescent area.

After 10 years of follow-up, there was no change in BCVA. However, SD-OCT of the right eye revealed hyperreflective foci within the EZ, located just above the temporal aspect of the optically empty space. In the left eye, hyperreflective foci were observed within the EZ, ONL, and the optically empty space (Figures 3D and 4). In addition, progression of the optically empty space was seen in both eyes, measuring 246 μm in width and 59 μm in height in the right eye, and 326 μm in width and 53 μm in height in the left eye. No change in ONL thickness (25 μm OD and 35 μm OS) and CMT (112 μm OD and 125 μm OS) was noted. Ultra-widefield autofluorescence of both eyes revealed a ring of hyperautofluorescence with a small central area of hypoautofluorescence in the fovea (Figure 5).

## Conclusions

We present a case of CNGA3-related achromatopsia demonstrating clear structural progression over time. To the best of our knowledge, this is one of the longest longitudinally OCT-documented cases of achromatopsia reported to date, with 10 years of follow-up.

A review of the literature reveals that fundus appearance and BCVA in patients with achromatopsia change minimally over time.^5,6^ Our case supports this observation, as neither fundus findings nor BCVA demonstrated changes over the 10-year follow-up period, with progression detected only on OCT imaging. This may explain why achromatopsia was previously thought to be a stationary condition in the pre-OCT era. Deterioration of cone function seems to be very slow or absent, as only minimal changes in BCVA have been documented during long-term follow-up.^6,7^ Moreover, as previously reported, ERG in achromatopsia shows severely reduced or extinguished cone responses from early childhood, without evidence of further deterioration over time.^8^ This suggests that cone function is compromised at very early stages. However, as seen in the present case and previous studies,^5,6^ early structural cone changes on SD-OCT may be subtle. Over time, progressive disruption of the EZ and the development of optically empty spaces become evident, indicating clear structural deterioration. Based on these findings, we hypothesize that at least in a subset of patients with achromatopsia, foveal cones show severely impaired function at birth despite relatively mild early structural changes on SD-OCT, with progressive deterioration over time.

In a 2-year prospective study published in 2014, Greenberg et al described an OCT-based staging system,^5^ and the findings observed in our case were consistent with their classification. At baseline (Figure 1), findings suggestive of stage 1 were observed, including a hyperreflective ELM and flattening with subtle discontinuity of the EZ. At 3 and 5 years of follow-up, features consistent with stages 2 (loss of EZ) and 3 (optically empty space) were observed (Figure 3, B and C). At the 10-year follow-up, evidence of possible RPE loss was noted, corresponding to stage 4 (Figures 3D, 4, and 5). Greenberg et al did not find a statistically significant correlation between the OCT-based staging system and the BCVA,^5^ which aligns with our findings, as there were no changes in BCVA despite clear structural deterioration.

The present case showed hyperreflective foci in the foveal area. The exact nature of these hyperreflective foci remains unknown; however, they may represent migrating mitochondria within the retina as a result of photoreceptor degeneration, similar to findings described in patients with outer retinal tubulation.^9^ An alternative explanation is the migration of RPE cells or melanin. A study correlating histology with SD-OCT in dry age-related macular degeneration showed that hyperreflective foci in the ONL corresponded to RPE cells.^10^ Furthermore, the authors reported that hyperreflective foci were frequently observed internal to areas that subsequently developed RPE thickening, supporting an association with RPE disturbance. Similarly, in our case, hyperreflective foci were observed adjacent to areas of mild choroidal hypertransmission on SD-OCT, presumably indicating early RPE disruption (Figures 3 and 4).

In our case, hyperreflective foci and disruption of the ELM were the first signs of disease progression observed on SD-OCT. Hyperreflective foci were present at least 3 years before detectable EZ changes, as seen in the 3-year follow-up image (Figure 3B). Furthermore, both the number and size of hyperreflective foci increased over time, in parallel with progressive EZ disruption and enlargement of the optically empty space. We propose that hyperreflective foci may represent a marker of disease progression in achromatopsia, similar to other hereditary retinal dystrophies such as retinitis pigmentosa and Stargardt disease. In these conditions, hyperreflective foci are thought to result from migrating RPE cells with subsequent development of RPE atrophy. In the present case, the exact origin of hyperreflective foci remains unclear and may reflect migrating RPE cells and/or mitochondria.

Our case demonstrated progressive enlargement of the optically empty space in both width and height over time, which is consistent with the findings reported by Triantafylla et al, who observed a statistically significant increase in the optically empty space without corresponding changes in CMT or ONL thickness.^6^ Similarly, Georgiou et al^7^ and Aboshiha et al^11^ reported no significant longitudinal changes in ONL thickness in patients with achromatopsia.

The SD-OCT changes in our case were mostly confined to the central fovea, with sparing of the parafoveal area, which is consistent with previous reports.^5,7,8^ Although the underlying mechanism has not been definitively established, we believe that this pattern is related to the rod-free zone in the center of the fovea. Given that achromatopsia primarily affects cones, a progressive generalized loss of these photoreceptors in the center of the fovea could result in EZ disruption and the formation of an optically empty space. In addition, a histopathology study showed the presence of cones in a fovea without a foveola, with a significant decrease in the cone density outside the fovea.^10^ The loss of cones in the parafovea and more peripheral areas of the retina may be compensated for by the presence of rods, which become the main contributors to the EZ band on SD-OCT. This may explain why EZ disruption and optically empty space formation are largely restricted to the rod-free zone. Furthermore, this compensatory mechanism may account for the relatively constant distance between EZ and the interdigitation zone with increasing eccentricity on SD-OCT scans. The lower concentration and progressive loss of cones in achromatopsia result in the absence of the cone outer segment tip signal on SD-OCT, and the typical shortening of cone outer segments with increasing eccentricity is not observed.^12^ Consistent with this, Georgiou et al, using confocal adaptive optics scanning light ophthalmoscopy in GNAT2-associated achromatopsia, showed lower mean cone densities not only at the foveal center but also at 190 μm, 350 μm, and 500 μm eccentricities compared with unaffected individuals.^7^

Our findings of hyperautofluorescence in the fovea are also consistent with achromatopsia being a progressive retinal disease. The loss of cone photoreceptors in the center of the fovea results in loss of macular pigment, which is responsible for the characteristic hypoautofluorescent signal seen in the fovea of healthy individuals. The small central area of hypoautofluorescence observed in our patient (Figure 4) likely corresponds to RPE disruption.

Overall, the patient was well supported, and we were able to refer her to a vision rehabilitation expert. She received guidance on reading strategies, including the use of magnified electronic text and audio-based reading options through accessible library services. Recommendations for daily activities included improving lighting conditions, using visual aids such as digital magnifiers and wearable devices, and exploring technologies that convert text to speech or provide live remote assistance. Safety and well-being were also addressed through counseling on safe food preparation techniques, strategies to enhance mobility confidence using peripheral vision, and access to peer support networks and disability resources.

In summary, we report a case of achromatopsia that, to the best of our knowledge, has the longest follow-up described in the literature. The progressive EZ disruption, appearance of hyperreflective foci, and development of optically empty space are consistent with findings from other studies that consider achromatopsia a progressive disease. Further studies utilizing adaptive optics technology may help elucidate the role of hyperreflective foci as a marker of cone degeneration and determine the source of EZ and interdigitation zone signals outside the rod-free area in patients with achromatopsia.
